# Mitophagy alleviates ischemia/reperfusion-induced microvascular damage through improving mitochondrial quality control

**DOI:** 10.1080/21655979.2022.2027065

**Published:** 2022-02-03

**Authors:** Dan Wu, Haizhe Ji, Wenjuan Du, Lina Ren, Geng Qian

**Affiliations:** a Department of Cardiology, The First Medical Center, Chinese People’s Liberation Army Hospital, Medical School of Chinese People’s Liberation Army, Beijing, China; b Department of Cardiology, The First Affiliated Hospital of Dalian Medical University, Beijing, China; c Laboratory of Radiation Injury Treatment, Medical Innovation Research Division, Chinese People’s Liberation Army General Hospital, Beijing, China; d Senior Department of Cardiology, The Sixth Medical Center of People’s Liberation Army General Hospital, Beijing, China

**Keywords:** UA, mitophagy, endothelial cells, cardiac microvascular injury, hypoxia/reoxygenation

## Abstract

The coronary arteries mainly function to perfuse the myocardium. When coronary artery resistance increases, myocardial perfusion decreases and myocardial remodeling occurs. Mitochondrial damage has been regarded as the primary cause of microvascular dysfunction. In the present study, we explored the effects of mitophagy activation on microvascular damage. Hypoxia/reoxygenation injury induced mitochondrial oxidative stress, thereby promoting mitochondrial dysfunction in endothelial cells. Mitochondrial impairment induced apoptosis, reducing the viability and proliferation of endothelial cells. However, supplementation with the mitophagy inducer urolithin A (UA) preserved mitochondrial function by reducing mitochondrial oxidative stress and stabilizing the mitochondrial membrane potential in endothelial cells. UA also sustained the viability and improved the proliferative capacity of endothelial cells by suppressing apoptotic factors and upregulating cyclins D and E. In addition, UA inhibited mitochondrial fission and restored mitochondrial fusion, which reduced the proportion of fragmented mitochondria within endothelial cells. UA enhanced mitochondrial biogenesis in endothelial cells by upregulating sirtuin 3 and peroxisome proliferator-activated receptor gamma coactivator 1-alpha. These results suggested that activation of mitophagy may reduce hypoxia/reoxygenation-induced cardiac microvascular damage by improving mitochondrial quality control and increasing cell viability and proliferation.

## 1. Introduction

Percutaneous coronary intervention (PCI) is a common revascularization method used to treat myocardial infarction. As PCI technology becomes increasingly advanced and standardized, more and more patients with coronary heart disease are accepting this treatment. However, some patients with precordial pain continue to experience chest pain after PCI, likely due to injuries to the arterioles, capillaries and venules of the coronary microcirculation. Coronary microcirculation injury is microvascular angina pectoris and/or no-reflow due to ineffective coronary perfusion, and has been estimated to occur in 5–50% of patients after acute revascularization. Microvascular damage is strongly associated with the development of heart failure; thus, it is important to understand the characteristics and risk factors of coronary microcirculation injury in coronary heart disease patients after PCI, in order to prevent heart failure and improve patients’ prognoses.

The specific mechanism of coronary microcirculation injury during PCI is not clear, but seems to include microvascular embolism, vascular endothelial cell inflammation, ischemia/reperfusion injury and insulin resistance caused by plaque rupture. Long-term hyperglycemia and smoking can impair the function of coronary vascular endothelial cells, leading to functional coronary microcirculation disorder. Stress during PCI enhances the inflammatory reactions of coronary microvascular endothelial cells, thus aggravating coronary microcirculatory dysfunction. Long-term hypertension contributes to myocardial hypertrophy, which promotes coronary microvessel degeneration. During or after PCI, embolism of ruptured plaques can further occlude the coronary microcirculation and thus limit the myocardial blood supply [1,2].

Several molecular mechanisms have been introduced to explain the cardiac microcirculatory damage after PCI, including oxidative stress, calcium overload, reduced vasodilator levels, enhanced inflammatory responses and an impaired anti-coagulation capacity [3–6]. Interestingly, these phenomena have been linked to mitochondrial damage; for instance, mitochondria are the sources of reactive oxygen species (ROS), so mitochondrial dysfunction is associated with oxidative stress [7,8]. Calcium recycling depends on adenosine triphosphate, which is undersupplied when mitochondrial function is impaired [2,9]. In addition, mitochondrial dysfunction-induced oxidative stress blunts the function of vasodilators such as nitric oxide and prostaglandins. Furthermore, uncontrolled mitochondrial damage activates cell death programs, which are followed by inflammatory responses. Therefore, protecting mitochondrial function is critical to sustain the myocardial microvascular integrity during or after PCI.

Mitophagy employs lysosomes to remove damaged mitochondria, and its benefits in endothelial cells have been widely described [10–12]. For example, mitophagy alleviates high-glucose-induced microvascular endothelial cell injury by suppressing oxidative responses [13]. In addition, mitophagy can inhibit oxidized low-density lipoprotein-induced endothelial cell apoptosis [14]. Mitophagy also attenuates oxidative stress-induced mitochondrial fission and apoptosis in endothelial cells [15]. Several studies have indicated that mitophagy can inhibit inflammatory responses and calcium overload [16–20]. In the present study, we asked whether mitophagy could protect mitochondrial function and thus maintain endothelial cell function.

## 2. Materials and methods

### 2.1 Cellular H/R model

Human umbilical vein endothelial cells (HUVECs) were randomly divided into three groups (n = 11): the control group, the H/R group and the UA group. For the H/R treatment, the original medium was discarded, low-glucose Dulbecco’s modified Eagle’s medium was added, and the cells were placed in a three-gas incubator (37°C, 5% CO_2_, 95% N_2_) for 4 hours of hypoxia [21]. The medium was then replaced with high-glucose Dulbecco’s modified Eagle’s medium containing 10% fetal bovine serum, and the cells were placed in an incubator (37°C, 5% CO_2_) for 4 hours of reoxygenation [8]. In the UA group, the cells were treated with UA (final concentration: 5 μg/μL) for 4 hours before H/R.

### 2.2 BrdU staining

HUVEC proliferation was evaluated using a BrdU cell proliferation detection kit (Guangzhou Saiyan Biotechnology Co., Ltd.) in accordance with the manufacturer’s instructions [22]. After being treated with the BrdU probe, the samples were treated with glycerin. Then, BrdU-positive cells were counted (200–500 cells) under the visual field of a fluorescence microscope [23].

### 2.3 Mitochondrial membrane potential detection

A mitochondrial membrane potential detection kit with JC-1 staining was used to determine the mitochondrial membrane potential of endothelial cells. After JC-1 staining, damaged cells exhibit green fluorescence, whereas normal cells exhibit red fluorescence [24]. A change from red to green fluorescence was used as an early indicator of a reduced mitochondrial membrane potential [25]. A fluorescence microscope was used to take pictures, and Image J software was used for fluorescence quantification.

### 2.4 Detection of oxidative stress

SOD activity, GSH levels and GPX levels were measured using enzyme-linked immunosorbent assay kits [26]. GSH and GPX levels were used to calculate the mitochondrial redox potential based on the Nernst equation [27].

### 2.5 CCK-8 assay

HUVECs were seeded on 96-well plates (100 cells at a density of 1 × 10^5^ cells/mL in culture medium) for 24 hours. Three wells were used for each group. At the beginning of reoxygenation, the original medium was discarded, and medium containing the CCK-8 solution (10 mg/mL) was added [28]. The cells were incubated for 4 hours, and the absorbance at 450 nm was measured on a microplate reader to reflect the cell viability [29].

### 2.6 Western blotting

HUVECs were seeded at a density of 1 × 10^5^ cells/mL, and were divided into three groups as described above (n = 3/group) when they were 80–90% confluent. After 4 hours of reoxygenation, radioimmunoprecipitation assay lysate (containing phenylmethylsulfonyl fluoride and a phosphorylase inhibitor) was added to each sample [30]. After being treated for 30 min on ice, the protein samples were collected and centrifuged at 4°C. The supernatants were obtained, and the protein concentration of each sample was determined using the bicinchoninic acid method [31]. The proteins were electrophoretically separated on gels and transferred to membranes. The membranes were blocked with 5% skimmed milk powder at room temperature for 2 hours. The gray values of the protein bands were determined using Image Pro Plus software. The gray value of the target protein was expressed relative to that of the internal reference [32]. The primary antibodies used in the present study were as follows: Bcl2 (1:1000, Cell Signaling Technology, #3498), Bax (1:1000, Cell Signaling Technology, #2772), caspase9 (1:1000, Cell Signaling Technology, #9504), c-IAP (1:1000, Cell Signaling Technology, #4952), GAPDH (1:1000, Cell Signaling Technology, #5174), Bad (:1,000; Abcam; #ab90435), Sirt3 (1:1000, Abcam, #ab86671), PGC-1α (1:1,000; Cell Signaling Technology, #2178).

### 2.7 Immunofluorescence

HUVECs were inoculated into six-well plates at a density of 1 × 10^5^ cells/mL, 3 mL/well. The cells were cultured in a 5% CO_2_ incubator at 37°C for 24 hours, and then were divided into three groups. After being reoxygenated for 4 hours, the cells in each well were washed with phosphate-buffered saline (PBS), fixed with 4% paraformaldehyde for 15 min, incubated with 0.3% TritonX-100 at room temperature for 30 min, washed with PBS and sealed with goat serum at 37°C for 30 min [33]. Then, the samples were incubated with antibodies at 4°C overnight. The cells were subsequently washed with PBS and incubated with fluorescent antibodies (1:200) at 37°C for 40 min. After being washed with PBS, the cells were incubated with 4′,6-diamidino-2-phenylindole (DAPI) at room temperature for 5 min. Lastly, the cells were washed with PBS, and the fluorescence intensity was measured using Image Pro Plus software [34].

### 2.8 Mitophagy detection

Mitophagy was detected through the mt-Kemia, which is a a ratiometric pH-sensitive fluorescent protein targeting into the mitochondrial matrix. A low-ratio mt-Keima derived fluorescence (543/458 nm) reports neutral environment, whereas a high-ratio fluorescence reports acidic pH. Thus, mt-Keima enables differential imaging of mitochondria in the cytoplasm and mitochondria in acidic lysosomes.

### 2.9 qPCR

qPCR was used to detect mRNA expression. Cells were isolated, stored in liquid nitrogen for 24 hours, and then stored at −80°C until analysis. The amplification conditions for qPCR were as follows: pre-denaturation at 94°C for 10 min to activate Taq polymerase, denaturation at 94°C for 15 sec, annealing at 60°C and extension for 60 sec [35]. After 45 cycles, target gene expression was detected using fluorescence qPCR (Applied Biosystems, USA). The cycle threshold (CT) value was calculated using the 2^−ΔΔCT^ method [36].

### 2.10 Statistical analysis

SPSS 16.0 software was used for statistical analysis, and GraphPad 5.01 software was used for data analysis. T tests were used to compare the groups, and p-values < 0.05 were considered statistically significant.

## 3. Results

### 3.1 Hypoxia/reoxygenation (H/R) injury induces mitochondrial dysfunction in endothelial cells

**Figure 1.**
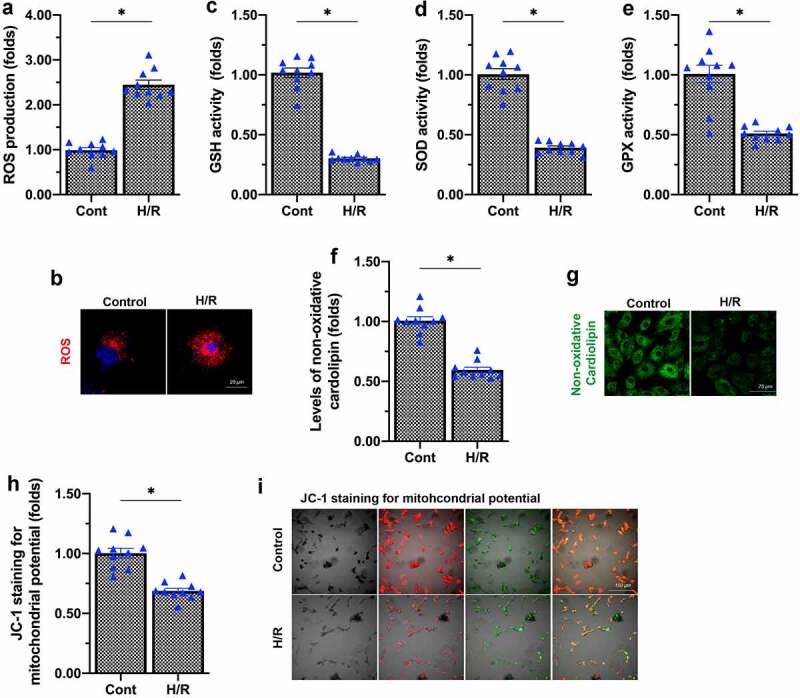
H/R injury induces mitochondrial dysfunction in endothelial cells. (a, b) An immunofluorescence assay was used to detect the alterations in mitochondrial ROS levels in response to H/R injury. (c-e) Enzyme-linked immunosorbent assays were used to detect GSH, SOD and GPX levels. (f, g) Immunofluorescence staining was used to detect non-oxidative cardiolipin. (h, i) The mitochondrial membrane potential was determined using JC-1 staining. *p < 0.05.

Mitochondrial damage is regarded as a key contributor to cardiac microvascular damage. Thus, we analyzed whether H/R injury induced mitochondrial damage in endothelial cells. ROS are primarily produced by mitochondria, and mitochondrial ROS overproduction is an early marker of mitochondrial dysfunction. We found that H/R injury augmented mitochondrial ROS generation in endothelial cells (Figure 1(a,b)).

Mitochondrial ROS overtly consume anti-oxidative enzymes, thus inducing the oxidation of mitochondrial membrane components, especially cardiolipin. We observed that H/R injury significantly downregulated anti-oxidative molecules such as glutathione (GSH), superoxide dismutase (SOD) and glutathione peroxidase (GPX) in endothelial cells (Figure 1(c-e)). Due to increased ROS production and a reduced anti-oxidative capacity, non-oxidative cardiolipin levels in endothelial cells were lower in the H/R injury group than in the control group (Figure 1(f,g)). Moreover, as a result of mitochondrial membrane damage, the mitochondrial membrane potential was reduced in H/R-treated endothelial cells, as evidenced by a reduced JC-1 immunofluorescence ratio (Figure 1(h,i)). These results illustrated that H/R injury induced mitochondrial dysfunction in endothelial cells.

### 3.2 H/R injury impairs endothelial cell proliferation and viability

**Figure 2.**
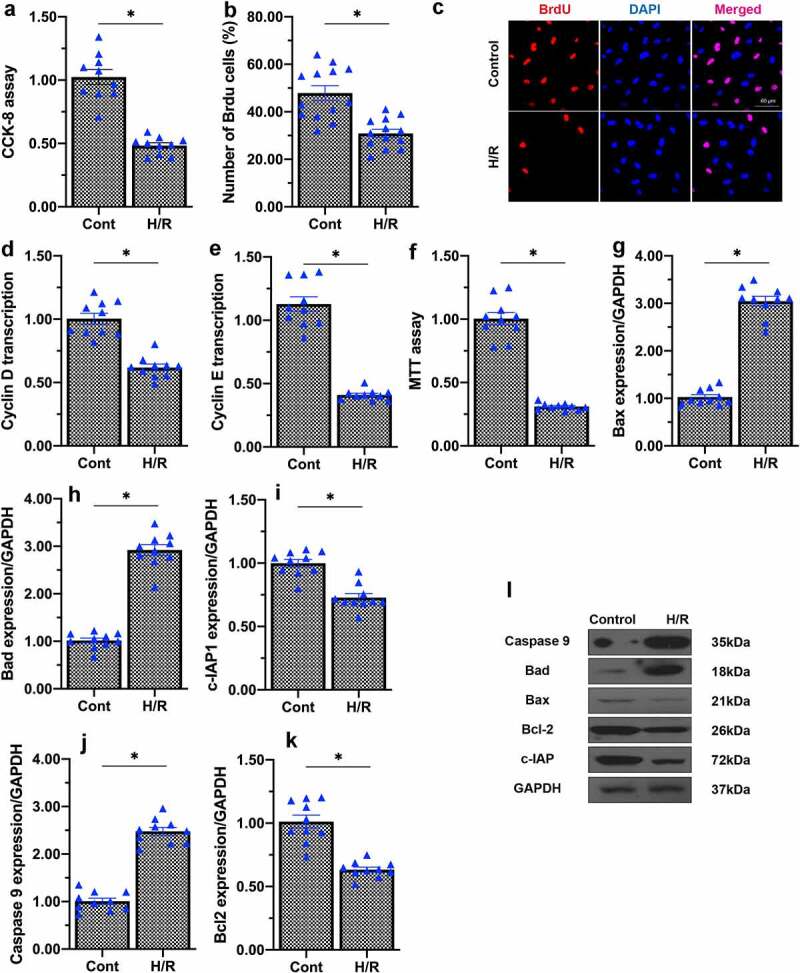
H/R injury impairs endothelial cell proliferation and viability. (a) Cell proliferation was evaluated using a CCK-8 assay. (b, c) Immunofluorescence staining was applied to observe the number of BrdU-positive cells. (d, e) qPCR was performed to assess the transcription of cyclin D and cyclin E. (f) Cell viability was measured using an MTT assay. (g-l) Western blots were used to observe the changes in Bax, Bad, caspase-9, Bcl-2 and c-IAP1 levels. *p < 0.05.

Mitochondrial damage hampers endothelial function [18,37]. In the cardiovascular system, the proliferation and mobilization of endothelial cells contributes to vascular tone and function. To assess cellular proliferation, we conducted a Cell Counting Kit 8 (CCK-8) assay, which indicated that H/R injury reduced the proliferation capacity of endothelial cells (Figure 2(a)). We also performed bromodeoxyuridine (BrdU) staining, which revealed that the number of BrdU-positive endothelial cells was significantly reduced upon H/R injury, confirming that H/R injury impaired endothelial cell proliferation (Figure (2b,c)). At the molecular level, H/R treatment reduced the transcription of proliferation-related genes such as cyclin D and cyclin E in endothelial cells (Figure 2(d,e)).

We then conducted a 3-[4,5-dimethylthiazol-2-yl]-2,5 diphenyl tetrazolium bromide (MTT) assay, which indicated that H/R injury reduced endothelial cell viability (Figure 2(f)). We also performed Western blots to detect apoptotic events in endothelial cells. Pro-apoptotic proteins such as Bax, Bad and caspase-9 were rapidly upregulated following H/R injury (Figure 2(g-l)). In contrast, anti-apoptotic factors such as Bcl-2 and cellular inhibitor of apoptosis 1 (c-IAP1) were significantly downregulated in H/R-treated endothelial cells (Figure 2(g-l)). These results demonstrated that H/R injury suppressed proliferation and induced apoptosis in endothelial cells.

### 3.3 Mitophagy activation sustains mitochondrial function in endothelial cells

**Figure 3.**
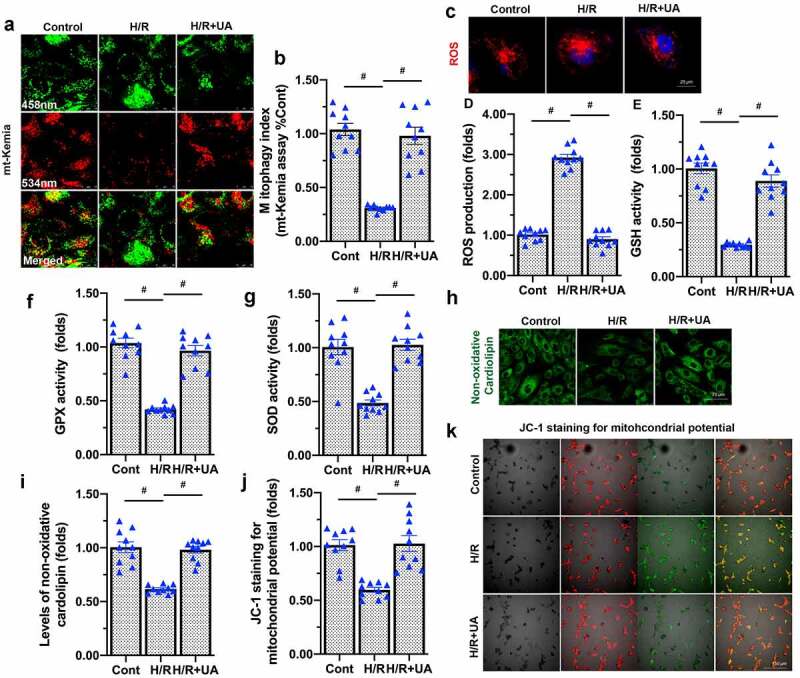
Mitophagy activation sustains mitochondrial function in endothelial cells. (a-b) mt-Kemia assay was used to evaluate mitophagy activity in under HR injury or UA treatment. (c-d) UA was added to activate mitophagy in endothelial cells. Then, an immunofluorescence assay was used to detect the alterations in mitochondrial ROS levels in response to H/R injury. (e-g) Enzyme-linked immunosorbent assays were used to detect GSH, SOD and GPX levels. (h-i) Immunofluorescence staining was used to detect non-oxidative cardiolipin. (j-k) The mitochondrial membrane potential was determined using JC-1 staining. #p < 0.05.

Considering that mitochondrial damage promoted endothelial dysfunction, we next examined whether mitochondrial protection via mitophagy augmentation could attenuate endothelial damage [38–40]. We pretreated endothelial cells with urolithin A (UA), an inducer of mitophagy. The mt-Kemia assay demonstrated that mitophagy activity was repressed by H/R injury and was restored by UA treatment (Figure 3(a,b)). Besides, our data further showed that UA reduced mitochondrial ROS levels following H/R injury (Figure 3(c,d)). UA supplementation also restored the anti-oxidative activity of GSH, SOD and GPX in H/R-treated endothelial cells (Figure 3(e-g)). As a result of the corrected redox response, non-oxidative cardiolipin increased to near-normal levels in UA-treated endothelial cells subjected to H/R injury (Figure 3(h,i)). UA also stabilized the mitochondrial membrane potential (a measure of mitochondrial damage) in H/R-treated endothelial cells (Figure 3(j,k)). These results demonstrated that mitophagy activation inhibited mitochondrial oxidative stress and sustained mitochondrial function in endothelial cells.

### 3.4 Mitophagy activation reduces H/R-induced endothelial apoptosis

**Figure 4.**
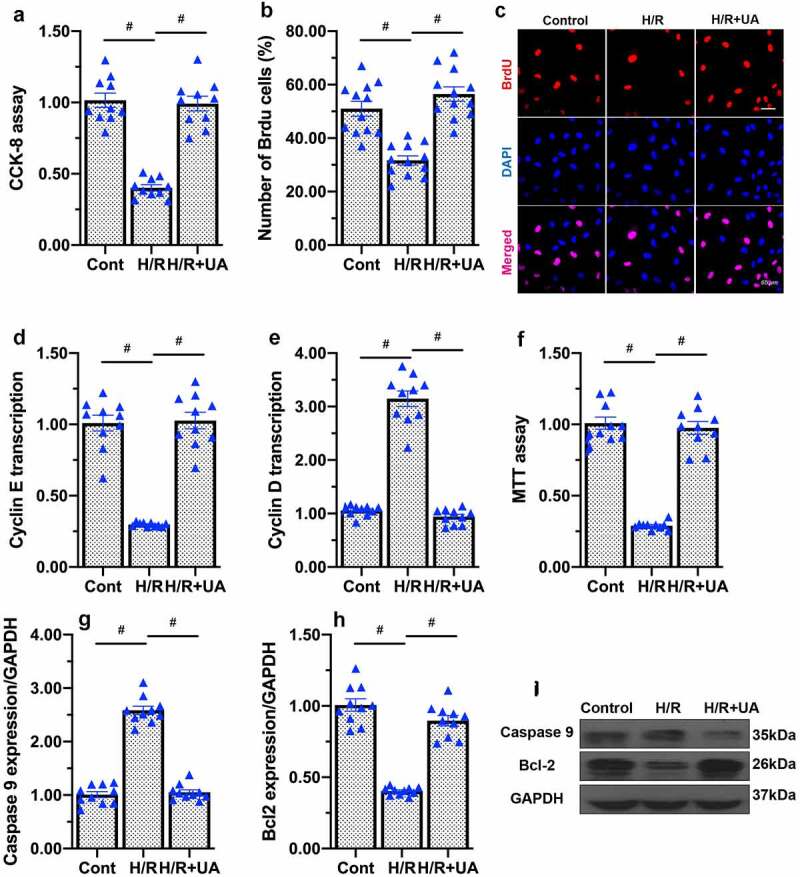
Mitophagy activation reduces H/R-induced endothelial cell apoptosis. (a) UA was added to activate mitophagy in endothelial cells. Cell proliferation was evaluated using a CCK-8 assay. (b, c) Immunofluorescence staining was applied to observe the number of BrdU-positive endothelial cells. (d, e) qPCR was performed to assess the transcription of cyclin D and cyclin E. (f) Cell viability was measured using an MTT assay. (g-i) Western blots were used to observe the changes in caspase-9 and Bcl-2 levels. #p < 0.05.

To analyze whether increased mitochondrial function could improve endothelial function, we assessed the proliferation and apoptosis of endothelial cells. A CCK-8 assay indicated that UA treatment restored the proliferation capacity of endothelial cells subjected to H/R injury (Figure 4(a)). Accordingly, the proportion of BrdU-positive endothelial cells was greater in the UA-treated H/R group than in the H/R group (Figure 4(b,c)). UA also enhanced the transcription of proliferative genes such as cyclin D and cyclin E in H/R-treated endothelial cells (Figure 4(d,e)). In addition to increasing the replicative capacity of endothelial cells, UA sustained the survival of these cells upon H/R injury, restoring their viability to near-normal levels (Figure 4(f)). This alteration may reflect the normalization of apoptosis-related proteins, as UA suppressed pro-apoptotic protein expression and upregulated anti-apoptotic factors in H/R-treated endothelial cells (Figure 4(g-i)). Based on these data, we concluded that mitophagy activation attenuated H/R-induced endothelial dysfunction by enhancing cell proliferation and inhibiting apoptosis.

### 3.5 UA improves mitochondrial quality control by activating mitochondrial fusion, inhibiting mitochondrial fission and promoting mitochondrial biogenesis

**Figure 5.**
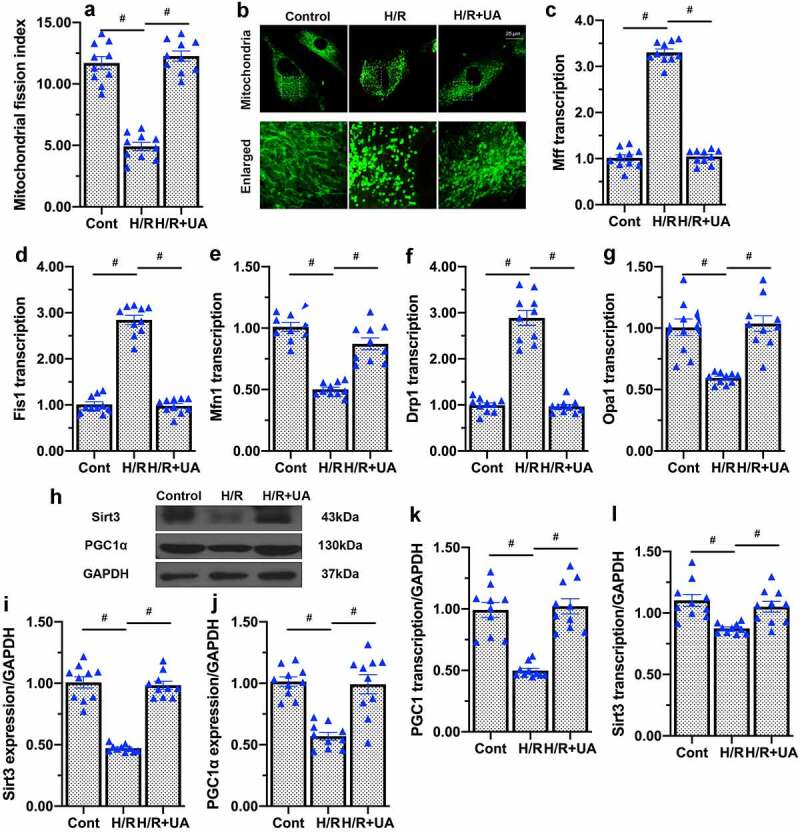
UA improves mitochondrial quality control by activating mitochondrial fusion, inhibiting mitochondrial fission and promoting mitochondrial biogenesis in endothelial cells. (a, b) Immunofluorescence was used to observe the mitochondrial morphology. (c-g) qPCR was used to analyze the transcription of *Drp1, Fis1, Mff, Mfn1* and *Opa1*. (h-j) Western blots were used to detect PGC1α and Sirt3 protein levels. (k-l) qPCR was used to detect the alterations of PGC1α and Sirt3 transcriptions in response to H/R injury or UA treatment. #p < 0.05.

The above data illustrated that mitophagy protects mitochondrial and cellular function in endothelial cells subjected to H/R injury, but the detailed mechanism remained unclear. Mitochondrial quality control measures such as mitochondrial fission, fusion and biogenesis allow mitochondria to adapt to stressful environments [41,42]. On the other hand, when mitochondrial quality control is impaired, mitochondrial metabolism is disrupted and mitochondria-related cell death is initiated. Therefore, we asked whether mitophagy increased endothelial cell mitochondrial function by normalizing mitochondrial quality control in the setting of H/R injury. Immunofluorescence analyses revealed that H/R injury promoted mitochondrial fragmentation, a result of increased fission and/or reduced fusion (Figure 5(a,b))[38]. Quantitative PCR (qPCR) analyses demonstrated that mitochondrial fission-related genes such as dynamin-related protein 1 (*Drp1*), mitochondrial fission 1 protein (*Fis1*) and mitochondrial fission factor (*Mff*) were upregulated in H/R-treated endothelial cells, while fusion-related genes such as mitofusin 1 (*Mfn1*) and optic atrophy 1 (*Opa1*) were downregulated (Figure 5(c-g)). Interestingly, UA prevented mitochondrial fission and improved mitochondrial fusion, ultimately reducing the number of fragmented mitochondria in H/R-treated endothelial cells (Figure 5(a,b)).

Mitochondrial biogenesis regenerates the mitochondrial population, and is primarily regulated by peroxisome proliferator-activated receptor gamma coactivator 1-alpha (PGC1α) and sirtuin 3 (Sirt3). PGC1α, which can be expressed in either the cytoplasm or the nucleus, regulates the transcription of genes involved in mitochondrial respiration. Sirt3 is primarily localized in mitochondria, and is an important regulator of mitochondrial redox biology and oxidative phosphorylation. After endothelial cells were subjected to H/R injury, Sirt3 and PGC1α were rapidly downregulated relative to the baseline; however, UA treatment upregulated PGC1α and Sirt3, thereby supporting mitochondrial biogenesis in H/R-treated endothelial cells (Figure 5(h-l)) both at the transcription and protein expression levels. These results indicated that UA normalized mitochondrial quality control programs (especially mitochondrial fission, fusion and biogenesis) in endothelial cells. Thus, mitophagy may protect mitochondrial and cellular function in endothelial cells by promoting mitochondrial quality control procedures.

## 4. Discussion

The coronary artery microcirculation is a cardiac microcirculatory system that includes small arteries, veins and capillaries [43,44]. Coronary microcirculatory dysfunction is a common complication of acute coronary syndrome and PCI, and is associated with no-reflow or a slow flow in the coronary artery. Coronary microcirculatory dysfunction is an important contributor to the occurrence, development and prognosis of cardiovascular disease [45,46].

The mechanism of coronary microcirculation injury is still unclear, but may involve a reduced coronary flow reserve. Female gender, diabetes, hypertension, previous myocardial infarction and pre-dilation time are the main clinical risk factors for coronary microcirculation injury after coronary artery disease (PCI). Diabetes is a risk factor for coronary heart disease because hyperglycemia increases vascular permeability, inhibits endothelial cell nitric oxide production and stimulates the release of vasoconstrictors, thus inducing abnormal vascular contractions. Hyperglycemia also increases extracellular matrix production in the microvessels, thus obstructing the lumen by promoting plasma protein deposition on the microvessel walls. Hypertension can aggravate the burden on small artery walls and induce microvascular structural remodeling under the influence of vascular growth factors. Long-term hypertension can damage the arterial endothelial structure, weaken endothelium-dependent vasodilation and increase vasoconstriction activity. In addition, hypertension can increase the left ventricular diastolic pressure, induce lumen stenosis in myocardial vessels due to mechanical compression, and reduce blood flow. Patients who have previously experienced myocardial infarction may have a heavy thrombus load, severe coronary microcirculation obstruction or poor myocardial perfusion, and thus are at increased risk for coronary microcirculation injury after PCI. In addition to the above clinical risk factors, ischemia has been found to initiate coronary microcirculation injury in basic studies. Coronary artery ischemia leads to endothelial acidosis, calcium overload, excessive inflammatory mediator and peroxide release, impaired endothelial cell barrier function and aggravated microvascular injury.

Endothelial cell-related coronary embolism, endothelial cell ischemia and/or reperfusion injuries can occur due to oxidative stress, and vasospasms can result from calcium overload [47,48]. With respect to endothelial cell-related coronary embolism, unstable atherosclerotic plaque rupture due to endothelial damage is associated with thrombosis, which leads to vascular mechanical obstruction and no-reflow after PCI [49]. Atherosclerotic plaque rupture increases tissue expression of tissue factor (TF, the initiation factor of coagulation) and releases it into the blood, thus contributing to acute thromboembolism. Regarding ischemia/reperfusion injury [50,51], long-term coronary artery ischemia induces endothelial cell swelling and interstitial edema, which may aggravate vascular occlusion. Reduced release of nitric oxide by injured endothelial cells impairs endothelium-dependent vasodilation, causing microvascular contraction. In addition to ischemic damage, oxidative stress is primarily induced at the stage of reperfusion due to neutrophil accumulation during the perfusion of fresh blood. More importantly, activated neutrophils release a large number of pro-inflammatory factors and ROS, thus increasing microvascular permeability, interstitial edema and extravascular compression. Lastly, dysfunctional endothelial cells release higher levels of vasoconstrictors such as 5-hydroxytryptamine and thromboxane A2, which reduce vasodilation and increase vasospasming.

Previous studies have examined the contribution of mitochondria to endothelial cell function during cardiac microvascular damage. Endothelial cell senescence has been identified as a major inducer of vascular aging [52]. Drugs targeting endothelial metabolic reprogramming have been found to ameliorate heart failure with preserved ejection fraction [53]. Endothelial cell-induced pulmonary hypertension is associated with endothelial mitochondrial damage, although the molecular basis is not fully understood [54]. High-glucose-induced endothelial dysfunction is associated with impaired mitochondrial function and increased apoptosis [55]. On the other hand, mitochondrial fitness highly sustains endothelium-dependent vasomotor function in endothelial cells [56]. In the present study, we identified mitochondrial damage as a feature of endothelial dysfunction [57–59]. Activating mitophagy protected the function of mitochondria and restored the function of endothelial cells by reducing their apoptosis, increasing their paracrine function and improving their proliferative capacity.

According to our data, UA treatment was associated with decreased mitochondrial damage, increased mitochondrial potential, elevated mitochondrial biogenesis, and inhibited mitochondrial apoptosis pathway. These effects may contribute to endothelial survival and enhance endothelial proliferation/viability. Compared with the previous findings [37,39,40,42], our study used UA to activate mitophagy and these results suggested that mitophagy agonist will be used in the clinical practice for the treatment of patients with myocardial microvascular injury. Despite the advances made in this study, several issues remain unaddressed. First, there is no unified standard for evaluating coronary microcirculation injury, especially in terms of mitochondrial function or mitophagy activity. The Index of Microcirculatory Resistance is the most accurate quantitative method to evaluate the degree of coronary microcirculation injury, and is not influenced by the degree of large artery stenosis or hemodynamics. However, this method is invasive and requires the use of vasodilators, so adverse drug reactions may occur and examinations may be costly. Second, there are not yet effective drugs to maintain mitochondrial function in endothelial cells. Nevertheless, our study has demonstrated that mitophagy and mitochondrial quality control are important for endothelial homeostasis.

## 5. Conclusion

Supplementation with the mitophagy inducer UA preserved mitochondrial function by reducing mitochondrial oxidative stress, stabilizing the mitochondrial membrane potential, inhibiting mitochondrial fission, augmenting mitochondrial fusion, and improving mitochondrial biogenesis, resulting into increased viability and proliferative capacity of endothelial cells.

## Data Availability

The datasets used and/or analyzed during the current study are available from the corresponding author on reasonable request.
